# Improvement in short‐term outcome over time, in a single center embarking on a canine mitral valve repair program using a structured multidisciplinary approach

**DOI:** 10.1111/vsu.14229

**Published:** 2025-02-28

**Authors:** Daniel J. Brockman, Thomas D. Greensmith, Matteo Rossanese, Alison Young, Sarah L. Carey, Adrian Boswood, Thaleia‐Rengina Stathopoulou, Carolina Palacios Jimenez, Nigel Cross

**Affiliations:** ^1^ Royal Veterinary College University of London London UK

## Abstract

**Objective:**

To describe a structured approach to the development of a mitral valve repair (MVR) program for dogs with myxomatous mitral valve disease (MMVD) and to document the change in short term outcome over time.

**Study design:**

Clinical prospective study.

**Animals:**

Dogs (*n* = 132) with MMVD undergoing MVR at a single hospital.

**Methods:**

Using a carefully structured multidisciplinary approach to patient selection, surgical treatment, and postoperative care that incorporated both “reflective” and “deliberate” practice, we embarked on an MVR program alongside our pre‐existing open heart surgery program. Data were gathered for the first 132 dogs undergoing MVR in this program (between July 2015 and November 2022). Short‐term survival was defined as dogs that were discharged from the hospital. The dogs were divided into four groups of equal size based on chronological order and the data within each group compared using descriptive statistics.

**Results:**

The breeds most represented were Cavalier King Charles Spaniels (CKCS) and Chihuahuas. The MMVD was categorized clinically as stage D in 36/132 (27%), stage C in 88/132 (67%) and stage B2 in 8/132 (6%) of affected dogs. Overall, 107/132 (81%) of the dogs survived to discharge. A consistent trend of improved short‐term outcome was observed, with survival rate of 22/33 in the first quartile, 27/33 in the second quartile, 28/33 in the third quartile, and 30/33 dogs in the fourth quartile.

**Conclusion:**

A structured organized approach by a team of consistent personnel resulted in an improvement in outcome over time. This improvement most likely reflected improvement in both technical and non‐technical skills within this complex sociotechnical environment.

**Clinical significance:**

What constitutes an acceptable performance for MVR in the dog has not been defined so the number of cases that constitute the “learning curve” during program development and the number of cases required in the “skills maintenance” phase remain unknown. The data reported here, however, describe the level of organizational commitment and the case throughput required to begin the process of establishing a successful MVR program and as such, should be useful to any team considering this therapy.

**Abbreviations**
MVR: Mitral valve repair
MMVD: Myxomatous mitral valve disease
CHF: Congestive heart failure
CPB: Cardiopulmonary bypass
MR: Mitral Regurgitation
ePTFE: expanded polytetrafluroethylene
ICU: Intensive care unit
FiO_2_
: fraction of inspired air that is oxygen
SQ: Subcutaneous
LMWH: Low molecular weight heparin
CKCS: Cavalier King Charles Spaniel
XCT: Cross clamp time
IVSH: Interventricular septal haematoma

## 1 INTRODUCTION

Myxomatous mitral valve disease (MMVD) is the most common heart disease in dogs.^1^, ^2^, ^3^ Progression of the disease is unpredictable and varies greatly among individual dogs but many will develop left‐sided congestive heart failure (CHF) that is ultimately fatal; with median survival of 1 year after the onset of failure, with medical treatment alone.^4^, ^5^ The preferred treatment for degenerative mitral valve disease in humans is mitral valve repair (MVR) or mitral valve replacement, depending on the circumstances.^6^, ^7^, ^8^, ^9^ Both MVR and mitral valve replacement have been reported in dogs.^10^, ^11^, ^12^, ^13^, ^14^, ^15^, ^16^, ^17^ The outcome for dogs undergoing mitral valve replacement using mechanical valves was poor with a median survival of 4.5 months after surgery, in the largest case series reported to date.^11^ Excellent results have been described with MVR in dogs with good short‐ and long‐term survival (median 38 months) along with improved quality of life for surgically treated dogs.^12^, ^13^, ^14^, ^15^, ^16^, ^17^, ^18^

Although open heart surgery in dogs is still in its infancy compared to human medicine, significant progress has been made in the last few years and the most recent consensus guidelines on MMVD now include open surgical repair as a treatment option for suitable candidates.^19^ It is important that the success reported by Kanemoto et al.,^12^ Uechi et al.^14^ and Matsuura et al.^16^ can be replicated by other centers and that information on the progression of skills and outcomes for “new” programs are reported, to help guide centers that are considering embarking on an MVR surgery program in the future. To be successful at MVR, the team delivering this therapy must: (1) agree on what constitutes a surgical candidate; (2) become competent at the technical aspects of cardiopulmonary bypass (CPB); such as circuit building and circuit priming, vascular access, and perfusion and cardioplegia techniques; (3) become familiar with preoperative and intraoperative assessment of the diseased mitral valve and thus determine the most appropriate repair; (4) become competent at anesthetizing dogs with severe mitral valve disease and the critical transition “on” and “off” CPB, in particular and (5) understand and meet the postoperative requirements of dogs recovering from this type of surgery. Arguably, this requires a high level of individual expertise as well as a high level of coordination and communication within a well‐functioning team.

The creation and maintenance of “high functioning” teams has been described and studied in several activity domains notably the aviation and military industries as well as a variety of medical disciplines.^20^, ^21^, ^22^ The principles that guide the conversion of a “team of experts” into an “expert team” were discussed by Bisby et al.^22^ and include: shared mental models, adaptability and resilience, clarity of role and responsibility, shared vision, dynamic leadership, cooperation and coordination, positivity and psychological safety. We hypothesized that a structured and well‐coordinated team approach that embraced these principles, would be required to obtain consistent results with MVR. The purpose of this study is to document the standard procedures that were adopted by a team of clinicians developing canine MVR capability and to document the change in short‐term outcome over time that was achieved using this approach. In addition, to describe and analyze the causes of fatality, where known, to help understand the interplay between “technical” factors and “disease” factors and how they influence outcome.

The aims of this study were to: (1) describe the standard practices adopted by a clinical team embarking on the development of an MVR program within a single institution; (2) describe the population of dogs that underwent MVR in respect of breed, age, gender and clinical stage of mitral valve disease; (3) report the short‐term outcome of MVR in dogs from this single institution and (4) report trends or alterations in the cause of mortality or suspected cause of mortality during the study period.

## 2 MATERIALS AND METHODS

### 2.1 Standard procedures and protocols within the MVR program

#### 2.1.1 Selection of suitable dogs

Dogs were screened for suitability at a “combined case conference” consisting of at least the primary surgeon, a member of the cardiology team, and the cardiothoracic case coordinators. Occasionally, the anesthesiologists and perfusionist would attend these meetings. These were held either face to face or online (during social distancing restrictions, in particular) and occasionally (for reasons of efficiency) in “sequence” (i.e., the cardiology team would review the dog data and make recommendations to the surgery team and case coordinators; who would meet separately). Data considered at the meeting for each dog included: signalment, echocardiographic data, echocardiographic video loops, results of blood tests, and thoracic radiographs. Dogs were considered candidates for MVR if they were free of any significant non‐cardiac disease and had a history of at least one episode of CHF (ACVIM^19^ stage C or D) or were considered to be in “advanced” stage B2. Advanced stage B2 was defined as severe left atrial enlargement and/or severe mitral regurgitation (MR) based on evaluation of MR vena contracta, mitral E wave velocity and presence of a flail leaflet.^23^ This determination was made or verified by one of the board‐certified cardiologists at our institution. All owners were carefully counseled as to the risks of surgery, and full owner consent was obtained.

#### 2.1.2 Surgery planning and coordination

Each dog scheduled for MVR was examined during the week prior to surgery and discussed individually at a meeting consisting of representatives from the cardiology service, surgery team, anesthesia team, transfusion medicine team and the critical care team, to ensure that the dog was stable, that all necessary resources were in place, and to confirm the surgical plan.

#### 2.1.3 Surgical procedure

The MVR procedure was adapted from techniques previously witnessed by one of the authors (DJB) and described for use in the dog.^12^, ^13^ Briefly, all dogs were anesthetized and underwent MVR under CPB and cardioplegic arrest, via a left fifth intercostal thoracotomy, following heparinization. The arterial access for the bypass circuit was via the left carotid or the right femoral artery. The venous drainage cannula was inserted into the right atrium either via the left jugular or the right auricular appendage. The perfusion circuit was primed with isotonic compound sodium lactate solution, heparin, mannitol, sodium bicarbonate and plasma. Cold (4°C) sanguinous cardioplegia solution was infused through a cannula in the ascending aorta following the application of an aortic cross clamp. Cardioplegia administration was repeated every 20 min or whenever electrical or mechanical cardiac activity was seen. The left atrium was opened, the mitral valve inspected and then repaired using artificial chordae tendineae of expanded polytetrafluroethylene (ePTFE) suture placed between the papillary muscles and the free edges of the valve leaflets. In addition, a pledgeted ePTFE purse‐string annuloplasty suture that extended around the mitral annulus from one fibrous trigone to the other was placed. The purse‐string annuloplasty was tightened and tied such that the annulus measured between 1.2 and 1.4 times the measured diameter of the aortic annulus (determined from the preoperative echocardiographic examination). The heart was closed and de‐aired and the repair evaluated using transesophageal echocardiography, before weaning fully off CPB, administration of protamine sulfate, and recovering the dog in our intensive care unit (ICU).

#### 2.1.4 Handover to the ICU team

This was performed by the anesthetist and the primary surgeon with the attending ICU clinician. One member of the surgery team continued nursing the dog until ICU technicians changed shifts at 20:00 h so as to be involved in the handover with the “night team.”

#### 2.1.5 Postoperative care

Once extubated, the dogs were put in an oxygen cage with an FiO_2_ of between 60% and 80% for the first few hours and this was reduced, as appropriate, based on serial blood gas analysis, with most dogs comfortable breathing “room air” by the morning after surgery. The thoracostomy tube was typically removed the following morning once drain output had decreased to <0.5 mL/kg/h consistently. Intravenous fentanyl was continued at 3 mcg/kg/h IV initially and adjusted according to perceived needs until the morning after surgery when it was discontinued and methadone 0.2 mg/kg IV every 4 h for 24 h was instituted, followed by buprenorphine 0.01–0.02 mg/kg IV or subcutaneously (SQ) for the following 24–48 h. Ropivacaine or bupivacaine were administered at 1.5 mg/kg through the thoracostomy tube every 6 h whilst it was in place. From 2015 to mid‐2019, dogs received a transfusion of type‐specific canine fresh whole blood (in an attempt to provide a limited number of platelets to the dog) after surgery, from mid‐2019 onwards, as the program became busier, type‐specific packed red cells were given, for convenience. The blood products were started once the protamine had been administered, or for those not tolerating protamine, once the surgical incisions had been closed. Further transfusions were based on clinical need and could include fresh whole blood, packed red blood cells, and/or fresh frozen plasma. Once intrathoracic bleeding had stopped, low molecular weight heparin (LMWH) therapy was initiated at 25 IU/kg SQ for the first dose, followed by 50 IU/kg SQ 12 h later for two doses (12 h apart) and maintained at 100 IU/kg SQ every 12 h for a further 5–7 days. In the latter part of the study period CKCS received a maintenance dose of 150 IU/kg SQ every 12 h. Aspirin (2–4 mg/kg orally every 24 h) and clopidogrel (2–4 mg/kg orally every 12 h for the first day then every 24 h) were initiated the day following surgery and maintained for 3 months. Anticoagulation therapy was adjusted as needed, by “suspending” LMWH treatment temporarily if clinical bleeding into the thorax, bladder or colon, for example, was seen. Complete blood cell count, biochemistry and an echocardiogram were repeated every 2–3 days during hospitalization or sooner if there were any concerns. Dogs were typically discharged at day 8–10 once blood work had normalized and the dogs were comfortable, with no clinical concerns.

#### 2.1.6 Debrief meeting

A meeting was held one or two days after each surgery to allow the care delivery team to discuss any adverse events and to highlight good practice from the surgery day. In addition, the dog's progress to date was discussed and a plan for the upcoming days was clarified at this time. In the case of a fatality, a separate meeting was convened once additional information had been gathered so that the team could discuss any areas for improvement in the light of either the known or suspected cause of death, when this was determined.

#### 2.1.7 Personnel

Several board‐certified cardiologists were involved in the selection of cases and the perioperative assessment of the heart and one cardiologist (VLF) was present throughout the study period. One surgeon (DJB) was present during all but three of the procedures, either as primary or supervising surgeon. Two cardiothoracic team coordinators (AY and SD) who provided intraoperative surgical assistance, postoperative nursing care and client communications, were present throughout the study period. The head perfusionist (NC) acted as senior perfusionist with oversight of the perfusion program throughout the study period but was not present at every surgical procedure. Perfusion services were provided by two clinicians trained in‐house (TG and AK) and on one occasion by an external commercial perfusion company. Several board‐certified anesthesiologists were involved in the program over the study period and one (CP) has had oversight of anesthesia provision for the entire study period. Several board‐certified critical care clinicians were involved in the aftercare but one (TG) was a consistent member of the team from mid‐2018 to the end of the study period.

### 2.2 Data collected

Medical records of dogs that had undergone MVR for the treatment of MMVD at the Royal Veterinary College, between July 2015 and November 2022 were examined. To be included in the study, dogs must have been managed entirely by our own team. Data taken from the medical records included: signalment, ACVIM stage of disease (based on the guidelines current at the time of surgery),^19^, ^24^ surgery date, cross clamp time (XCT) and cause of death where known, or suspected cause of death. Survival was defined as: survived to be discharged from the hospital.

### 2.3 Statistical analysis

Descriptive statistics (median, and range) are used for the age, bodyweight, XCT for the whole study population and then for each chronological quartile of 33 dogs. Breed frequency is listed for the whole group and for the most common breeds, for each quartile. The survival data is expressed graphically with 95% confidence intervals. Dogs were allocated an “operative rank” representing the order in which they were operated on from one for the first dog to 132 for the last dog. The mean rank of the non‐surviving dogs was compared to the mean rank of the surviving dogs using a Mann Whitney test. A *p*‐value < .05 was considered statistically significant.

## 3 RESULTS

### 3.1 Population

A total of 132 dogs that underwent MVR for MMVD at our center met the criteria for inclusion in the study. Median age was 9.9 years (range, 1.8–14); median bodyweight 6.75 kg (range, 2.3–28.6). Castrated male dogs represented 66/132 (50%), spayed females were 45/132 (34%), entire male dogs were 16/132 (12%) and entire female dogs were 5/132 (4%). A total of 15 different breeds were represented with CKCS the most common breed at 31/132 (23.5%), followed by Chihuahuas at 29/132 (21.9%) and mixed breed dogs at 28/132 (21%) (Table 1). As a subset of the mixed breed dogs, poodle cross breed dogs were 13/132 (10%) of the total population. The median cross clamp time was 73 min (range; 40–165) with median XCT of 77, 66, 71 and 81 for quartiles 1–4, respectively. The MMVD was categorized clinically as stage B2 in 8/132 (6%), stage C in 88/132 (67%) and stage D in 36/132 (27%) of affected dogs.

**TABLE 1:** Breed distribution of dogs undergoing MVR.

Breed	Number	Percentage (%)
CKCS	31	23.5
Chihuahua	29	22.0
Cross Breed	28	21.2
(Poodle X)	(10)	(9.8)
Maltese	11	8
Pomeranian	6	4.5
Dachshund	5	6.7
Shih Tzu	4	3.0
Yorkshire Terrier	4	3.0
Border Collie	4	3
JRT	3	2.2
Havanese	3	2.2
Pekingese	2	1.5
Beagle	2	1.5
Griffon	1	0.7
Norfolk Terrier	1	0.7

Abbreviations: CKCS, Cavalier King Charles Spaniel; JRT, Jack Russell Terrier; MVR, mitral valve repair; X, cross.

### 3.2 Survival

Overall, 107/132 (81%) of the dogs survived to discharge. The 25 dogs that died are summarized in Tables 2 and 3. Seven dogs died in the operating room either because of failure to wean from CPB, uncontrollable hemorrhage or refractory hypotension once weaned from CPB. One of these dogs had developed a large interventricular septal hematoma (IVSH). A total of 15 dogs underwent cardiopulmonary and/or respiratory arrest in ICU and five of these dogs underwent cardiopulmonary arrest following a period of refractory hypotension. Four dogs had acute neurological deterioration with signs consistent with intracranial vascular disease (stroke) and two of these dogs had ultrasonographic evidence of thrombus in the left heart, and one had an IVSH. Three of the dogs that died in ICU had intrathoracic hemorrhage that resulted in hypotension causing cardiopulmonary arrest one each at 12, 24, and 48 h after surgery. Two dogs died with evidence of multiple organ dysfunction along with hypotension and one brachycephalic dog died in ICU because of obstruction of a tracheostomy tube. Three dogs underwent acute sudden death in the wards (presumed arrhythmic) and one of these dogs was being treated with mexiletine for occasional ventricular premature complexes presumed to be secondary to a myocardial infarct following surgery (on the basis of ECG changes and echocardiographic evidence of left ventricular free wall akinesis). One of the eight dogs classified as B2, 15 of the 88 dogs classified as stage C, and nine of the 36 dogs classified as stage D, died.

**TABLE 2:** Summary of fatalities in chronological order.

	Breed	Sex	Age	BW (kg)	Stage	XCT	Additional notes
First quartile							
	Dachshund	MN	7	14.5	Stage D	165	CPA in ICU
	CKCS	MN	7	9.5	Stage D	139	Failure weaning CPB
	CKCS	MN	10	10	Stage C	112	Failure weaning CPB ‐suspect MI
	CKCS	FS	7	10.2	Stage D	140	Failure weaning CPB
	CKCS	MN	6	10.6	Stage D	151	CPA in ICU
	Shih Tzu	ME	9	6.7	Stage C	120	CPA in ICU ‐ poor pulmonary function based on ABG
	Chihuahua	MN	7	3.7	Stage C	57	CPA in ICU ‐neurological‐altered mentation
	Border Collie	FS	6	16.1	Stage‐C	90	Failure weaning CPB
	CKCS	ME	10	8.9	Stage D	80	CPA in ICU ‐ LA and valve thrombus + neurological (brainstem) signs
	Pomeranian	MN	8	4.6	Stage C	69	CPA in ICU ‐ IVSH
	Jack Russell Terrier	FS	11	3.6	Stage D	68	CPA in ICU ‐ poor pulmonary function based on ABG
Second quartile							
	Havanese	MN	10.5	5.7	Stage D	93	CPA in ICU ‐ MODS
	Dachshund	ME	10	14.4	Stage D	81	CPA in wards ‐ suspect MI
	Bruxellois Griffin	MN	9	4.2	Stage C	53	Respiratory arrest in ICU ‐ BOAS and trach tube obstruction
	Maltipoo	FS	11	4.2	Stage‐D	84	CPA in OR post weaning
	Shih Tzu	MN	4.5	6	Stage C	65	CPA in wards known to have MI postoperatively
	Chihuahua	FS	10	3.2	Stage C	65	Neurological complications/stroke ‐PTS
Third quartile							
	Pomeranian	FS	8	3.6	Stage C	79	CPA in ICU ‐Neurological complications/stroke
	Chihuahua	FS	10.5	2.3	Stage C	70	Failure weaning CPB
	Chihuahua	FS	13	2.6	Stage C	85	CPA in OR ‐ perioperative hemorrhage suspect RA hole
	Chihuahua	FS	9	3.1	Stage C	60	CPA in ICU ‐ MI in cardiology. V. fib in ICU
	Chihuahua	FS	8	6.3	Stage B2	87	CPA in ICU ‐intrathoracic hemorrhage post owner visit
Fourth quartile							
	Shih Tzu	FS	8	6.8	Stage C	77	CPA in ICU postoperative hemorrhage
	Maltese	FE	10	3.3	Stage C	81	CPA in ICU ‐ MODS
	Chihuahua	FS	9.3	5.2	Stage C	70	CPA in ICU ‐hemorrhagic shock following removal of chest drain and subcutaneous bleeding

Abbreviations: ABG, arterial blood gas; BW, bodyweight; CKCS, Cavalier King Charles Spaniel; CPA, cardiopulmonary arrest; CPB, cardiopulmonary bypass; FE, female entire; FS, female spayed; ICU, intensive care unit; IVSH, interventricular septal hematoma; LA, left atrium; ME, male entire; MI, myocardial infarct; MN, male neutered; MODS, multiorgan dysfunction syndrome; OR, operating room; PTS, put to sleep; RA, right atrium; V.fib, ventricular fibrillation; XCT, cross clamp time.

**TABLE 3:** Comparison of measured parameters between dogs that survived and dogs that did not survive to discharge.

	Breed CKCS/Chihuahua	Median age (years) (range)	Median BW (kg) (range)	Clinical stage: B2/C/D	Median XCT (minutes) (range)
Survived (*n* = 107)	26/22	10 (1.8–14)	7.1 (2.4–28.6)	7/53/27	71 (40–120)
Died (*n* = 25)	5/7	9 (4.5–13)	5.7 (2.3–16)	1/15/9	81 (53–165)

Abbreviations: BW, bodyweight; CKCS, Cavalier King Charles Spaniel; XCT, cross clamp time.

### 3.3 Change over time

**FIGURE 1:**
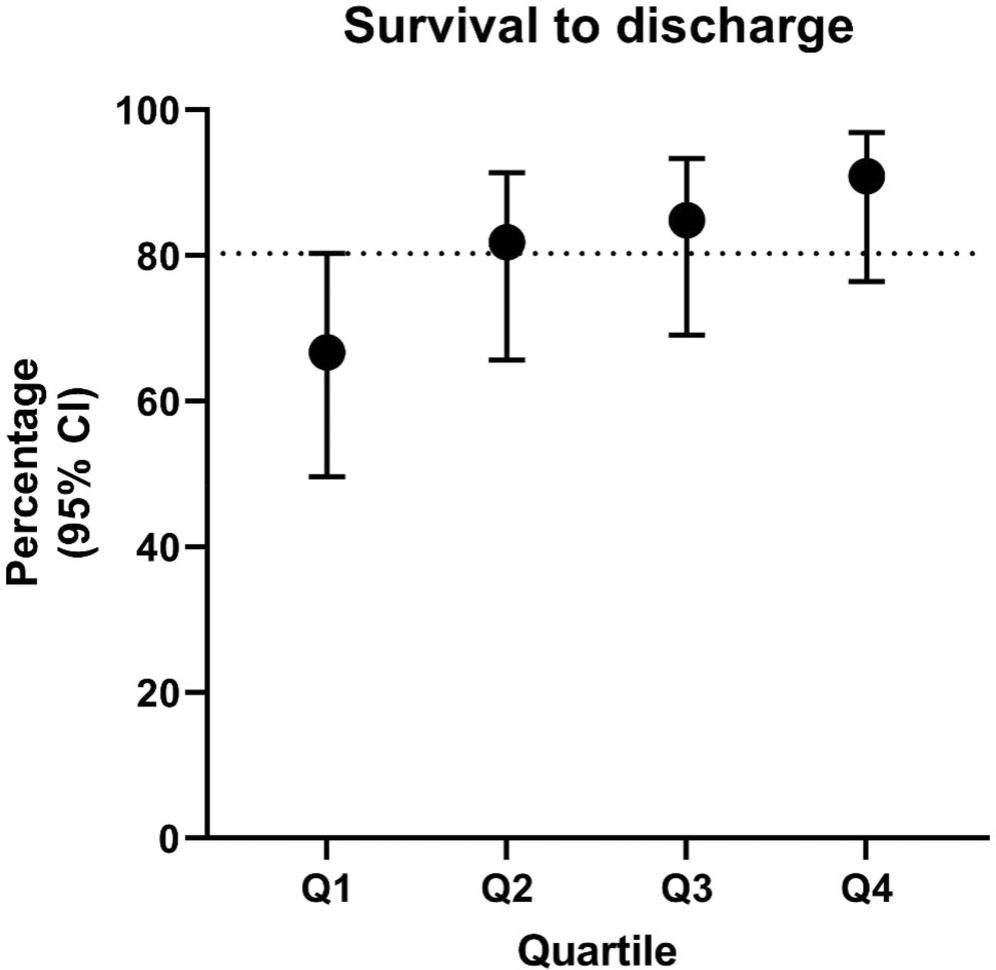
Percentage survival to discharge for each successive quartile, with 95% CI. The bold circle represents the proportion that survived to discharge in each quartile. The error bars indicate the 95% CIs of the estimate of the proportion.

**FIGURE 2:**
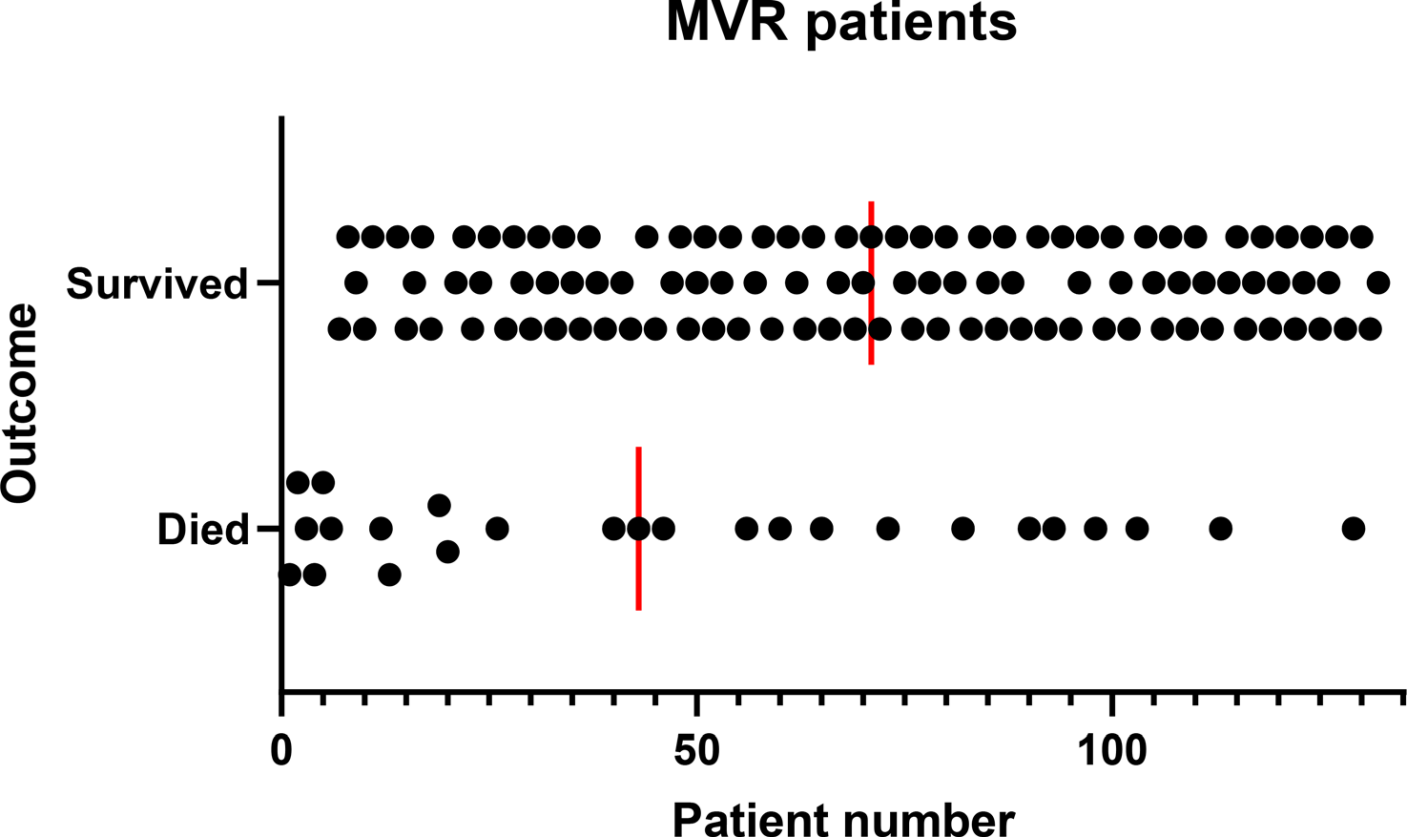
Operative rank of mitral valve repair dogs is illustrated according to whether or not they survived. The medians of the two groups are illustrated by the vertical red bars. MVR, mitral valve repair.

A total of 33 dogs were included in each quartile group. The comparison of breed frequency for the two most common breeds (CKCS, Chihuahua), age at the time of surgery, bodyweight, clinical stage and cross clamp time were similar and are summarized in Table 4. In the first quartile, 22 of 33 dogs survived to discharge, in the second quartile, 27 of 33 dogs survived to discharge, in the third quartile, 28 of 33 dogs survived to discharge and in the final quartile, 30 of 33 dogs survived to discharge. The change in survival between each quartile, with 95% confidence intervals are illustrated in Figure 1. The mean operative rank of MVR dogs comparing the dogs that survived and the dogs that died is illustrated in Figure 2. With the median rank of each group illustrated by the vertical red bars. This means that the later in the study period a dog was treated (i.e., higher operative rank number), the higher the probability of survival. The difference between the mean operative rank of the dogs that died and of the dogs that survived was significant.

**TABLE 4:** Comparison of each quartile group.

Variable	Overall	Quartile 1 (*n* = 33)	Quartile 2 (*n* = 33)	Quartile 3 (*n* = 33)	Quartile 4 (*n* = 33)
Breed CKCS/Chihuahua	31/29	11/7	4/8	7/8	8/6
Median age (years) at surgery (range)	9.9 (1.8–14)	8 (5–12)	10 (4.5–12.5)	10.5 (1.8–14)	10 (5.5–12.5)
Median BW (kg) at surgery (range)	6.75 (2.3–28.6)	6.7 (2.4–23)	5.7 (2.5–21.5)	6.8 (2.3–20.3)	7.4 (2.5–28.6)
Clinical stage: B2/C/D	8/88/36	1/22/10	1/23/9	2/23/8	4/20/9
Median XCT (minutes) (range)	73 (40–165)	77 (55–165)	66 (40–93)	71 (53–108)	81 (60–120)
Dogs surviving to discharge	*n* = 107	*n* = 22	*n* = 27	*n* = 28	*n* = 30

Abbreviations: BW, bodyweight; CKCS, Cavalier King Charles Spaniel; XCT, cross clamp time.

## 4 DISCUSSION

This study describes the organizational approach we adopted throughout the MVR study period and which we continue to use. In addition, we describe the improvement in outcome over time achieved by a team learning how to perform MVR at a single institution. The 132 dogs in the study reported here were the first 132 MVR operations performed by this team since starting in 2015. Several members of the team had, however, been involved with open heart surgery under conditions of CPB, since the program was restarted at our institution in 2005; the results of some of which have been reported previously.^25^, ^26^ Consequently, the team was already somewhat familiar with the process of open‐heart surgery having gained experience in 22 dogs undergoing a variety of procedures, prior to initiating the MVR program. The signalment data presented here were chosen to demonstrate that the population remained consistent throughout the study period and between quartile groups. It is difficult to compare the population of dogs in our study with other publications in this field because other studies selectively described MVR in either “toy breed” dogs^12^, ^14^ or were selective for larger breeds of dog.^10^ Within each quartile group, age, breed, gender, bodyweight and clinical stage frequency distributions were similar; suggesting that there was no case selection bias. We have shown a gradual improvement in short‐term outcome over time, with significantly increased odds of survival towards the end of the study period. Neither subjective nor objective evaluation of the “quality” of the MVR are reported in the present study, although the owner assessed “quality of life” of a subset of these dogs has already been described^18^; and those data suggested that the repair was “good enough” in that group of dogs. In an experimental study of MVR in a canine model of mitral valve incompetence, Nagatsu et al. concluded that the indices of cardiac performance would return to normal (i.e., levels equivalent to prior to the deliberate creation of mitral incompetence) providing the repair achieved a left ventricular regurgitant fraction of 30% or less.^27^ On this basis and concerned that trying to achieve a “perfect” repair might be detrimental to the chance of any individual dog surviving the attempt, our philosophy at the outset of this program was that the most desirable outcome for all “stake‐holders” was an imperfect valve repair in a dog that survived treatment, rather than a perfect valve repair in a dog that did not survive. It remains critical that, in the future, subjective and objective measures of the quality of valve repair are combined with long‐term outcome data, to determine optimal repair techniques, and repair assessment parameters. This may be possible now that several centers have developed sufficient technical competence with MVR.

Cross clamp time was originally included in the data with the anticipation that it would be a “surrogate” indicator of overall improvement in technical performance and as such, there would be an initial reduction in XCT and then it would stabilize. However, although XCT reduced initially, it increased again in the fourth quartile, which was a surprise. In human studies, where cardiopulmonary bypass techniques and protocols are “stable and established,” a long XCT is independently associated with increased morbidity and mortality in patients with equivalent risk‐adjusted ratings in some studies^28^, ^29^ but not in others.^30^ Presumably, longer XCT as a single variable may correlate either with the complexity of the disease or repair, and thus a reflection of a “patient factor,” or as a result of an adverse technical event or intraoperative mistake and represent a “technical factor.” We did not attempt to analyze the cause of longer XCT so we must be cautious regarding the interpretation of these data in this study. The longer XCT in the fourth quartile could have been secondary to the “slow down” of case throughput during the COVID‐19 pandemic “lockdown” period causing deterioration of the technical skills required to perform MVR. It is also possible that, over time, the surgery team developed an improved understanding of the valve pathology and thus the “repair” needed and so, confident in other aspects of the process, were prepared to spend additional time ensuring that the repair was good. The latter might become more apparent if the quality of repair were to be analyzed.

Our interpretation of the pattern of fatalities following MVR is that they could be categorized as: (1) technical challenges during surgery that resulted in death in the operating room or death in the immediate postoperative period (failure to wean, myocardial infarction [MI], IVSH, acute lung injury); (2) technical challenges with postoperative management and anticoagulation, in particular (stroke‐like events, postoperative MI and intrathoracic hemorrhage after removal of chest drain) and (3) late complications such as multiorgan failure. We appreciate that there may be different interpretations and that without definitive post‐mortem data, our interpretation remains speculative. During the study period, however, it became clear that dogs with MMVD undergoing MVR are a very “unforgiving” population and that challenges/complications experienced at any point during the attempt at MVR had, subjectively at least, a multiplicative rather than additive effect on the overall risk of failure. This “multiplicative” effect was described by de Leval et al. in a multicenter study evaluating the human factors in pediatric cardiac surgery.^31^ In studies of surgical outcomes in human medicine, the population of patients is typically “risk adjusted” and the technical aspects (non‐surgical operating room procedures and postoperative care) are assumed to be uniform so that the effect of the surgeon/surgery team can be isolated as a variable for analysis.^32^ It is currently not possible to perform a similar analysis in veterinary medicine. In the study reported here we have described a shift from a relatively high mortality rate to a much lower one within a “complex sociotechnical system” that could be considered analogous to human cardiac surgery. We consider that our team is still in the “skills acquisition” phase which is now overlapping with the “skills maintenance” phase of treatment delivery.

In human medicine the “surgeon volume” and “surgery team volume” that is required for a consistent outcome for complex procedures has been studied. Threshold volume of 65 cases per hospital per year and 15 cases per surgeon per year were identified in a study examining 30‐day readmission rates for total hip replacement (THR) in man.^33^ In human MVR, more than 20 procedures per year per surgeon was associated with better outcomes in one study^32^ and more than 30 procedures per year were recommended for surgeons performing thyroidectomy for differentiated thyroid carcinoma.^34^ Hayes et al. adapted the cumulative summation (CUSUM) technique to assess the learning curve for canine THR and concluded that approximately 44 THR operations constituted the “learning curve” accepting a 10% complication rate for supervised trainees.^35^ Extrapolating from these studies, any veterinary center considering MVR surgery needs to commit a team to: (1) working in a complex sociotechnical system (that may be different from their current customs and practice); (2) accrue sufficient case experience to become proficient and (3) commit to performing a relatively high number of procedures annually, to maintain the requisite skills. Whilst the number of operations required to become and remain proficient at canine MVR have not yet been established, now that centers have developed expertise, the ongoing monitoring of performance of new trainees should help us understand this better. The challenges of teaching these techniques in the veterinary setting, however, may be analogous to those recognized in teaching pediatric cardiac surgery in man, and highlighted by Winlaw et al. in their review “Leadership, surgeon well‐being and non‐technical competencies of pediatric cardiac surgery” which points to the particular difficulty senior surgeons might have “relinquishing control” to trainees in such an “high stakes” environment that has such a low tolerance for mistakes.^36^

We have documented a substantial improvement in short‐term survival over time with significantly increased odds of survival in the most recent quartile. To achieve good short‐term results with MVR, we had to create a well‐functioning team that were united around the desire to develop this treatment. Throughout the course of the study period, the team adopted customs and practices to enable the development of technical competence in the many different key tasks required to perform MVR in a reliable, reproducible way. Although the members of the team inevitably changed occasionally, several members were consistent throughout the study period providing important continuity and consistency of “team memory”. Every subgroup within the team (cardiology, surgery, anesthesia and critical care, transfusion medicine, perfusion medicine) had to acquire additional technical skills within their own discipline as well as adopting habits that would enable a competent team performance. The principles that allow an individual to develop into an “expert performer,” specifically the engagement in effortful activities (deliberate practice), were first put forward by Ericsson et al.^37^ and have subsequently been discussed in the field of psychology, in respect of “expert team performance” in the context of medical, military and aviation industry domains.^20^, ^21^ The role of deliberate practice (defined as “engagement in structured activities created specifically to improve performance in a domain”), along with “genetic factors” and “scientific training methods” in the acquisition and maintenance of expert performance in medicine and related fields, were further discussed by Ericsson,^20^ Burke et al.^21^ and Ericsson and Harwell.^38^ Burke et al. described guidelines for effective team training^21^ and as previously mentioned, eight principles of expert team performance were proposed by Bisbey et al.^22^ The initial challenge associated with developing an MVR program was the lack of specific training for our team. One member of the team (DJB) had visited two centers in Japan performing MVR (Chayagasaka Animal Hospital–Nagoya, Japan and Jasmine clinic–Yokohama, Japan) and the team from the Jasmine clinic traveled to the UK to operate in our facility on five occasions to perform seven MVR procedures in total, allowing our team to observe the technical and non‐technical skills they employed during each procedure. Essentially, this was our opportunity to perform the “task identification and analysis” that Burke et al.^21^ suggest should be the first step. The actual technical skills required for the surgical repair were particularly challenging to learn directly; and with MVR in dogs still in its infancy worldwide, there were/are no established training centers. For our team, learning technical skills by “deliberate practice” and through reflective discussion after each surgical event, helped unite us around this goal. In addition, the open and willing intellectual exchange of experiences, within the small “community” of others working in this field worldwide, increased the speed with which progress was made for everyone. During the period of study reported here, we integrated the steps necessary to maintain a competent team performance into our normal working lives so that each treatment event could be treated as “deliberate practice” with appropriate reflection and the identification of good practice and areas for improvement. Ideally, new centers that wish to develop an MVR program will begin the process by training their team at one of the centers that is already established.

The improvement in short‐term outcome over time was achieved on similar dog populations in each quartile and so is likely due to improvement in both technical and non‐technical performance of the team. The team were, and still are, in the process of making improvements and refinements to our procedures, processes and protocols; to further reduce the risk of harm to the dogs. Early in the development of our program, we sought the services of a perfusionist actively working in a human pediatric cardiac surgery team and contracted to us on a case‐by‐case basis. This approach reduced/controlled the additional “risk” to the dogs, associated with an inexperienced perfusionist performing perfusion infrequently. Ultimately, case throughput was sufficient to allow two perfusionists to be trained “in house” and to become technically competent at the skills required. Once each subteam had developed competence in the “foundation skills and procedures,” further development depended on the refinement of these by each group. This highlights the importance of having consistent personnel and the value of regular discussions around each individual dog so that opportunities for small incremental changes can be identified and made. The idea of constantly looking for small improvements in each individual modifiable contributor, in the quest for improved overall performance through the “aggregation of marginal gains,” is relevant to many domains including sport, the military and business practice as well as in medicine.^39^, ^40^, ^41^ Our team have adopted the same philosophy as we look for improvement and we continually make small adjustments, as we strive for consistency of outcome. Thus, there have been many small changes throughout the study period. Our report might seem like a simplification of the available data, but deeper statistical interrogation of the myriad of variables “in play” associated with such a variety of different causes of fatality, would not be meaningful until we have reached “steady state” with the variables under our control. In short, the biggest risk at the beginning of the study period was the technical skill set of the team; as we gain technical stability, disease factors may become the greater risk. Now we have reached this point, although there is still much to learn, our technical skills are more “stable” and there are now very few “adverse events.” Because of the low frequency of adverse events, it will require much higher numbers of dogs in order to make any useful comment about either technical or non‐technical (disease‐related) risk factors for mortality among dogs undergoing MVR.

## AUTHOR CONTRIBUTIONS

Brockman DJ, BVSc, Diplomate ACVS, Diplomate ECVS: Conception of MVR program, acquisition, analysis and interpretation of data, drafting and editing the final manuscript. Greensmith TD, BVetMed, MVetMed, Diplomate ACVECC, Diplomate ECVECC, Rossanese M, DVM, SPSA, CertAVP, MSc, Diplomate ECVS, Young A, RVN, Diplomate AVN (Surgery), VTS (Surgery), Carey SL, RVN, Stathopoulou T‐R, DVM, MVetMed, Diplomate ECVAA, Palacios Jimenez C, DVM, PhD, Diplomate ECVAA and Cross N, PGDip, HSM, FCCP: Acquisition of data and revision of the manuscript. Boswood A, MA, VetMB, Diplomate ECVIM‐CA (Cardiology): Analysis of data and revision of the manuscript.

## FUNDING INFORMATION

Fellowships were made possible thanks to donations from the Tailwaggers Club to the Royal Veterinary College.

## CONFLICT OF INTEREST STATEMENT

The authors declare no conflict of interest related to this report.
